# Interesterification of Glyceryl Trioctanoate Catalyzed by Sulfonic Silica-Based Materials: Insight into the Role of Catalysts on the Reaction Mechanism

**DOI:** 10.3390/ma16145121

**Published:** 2023-07-20

**Authors:** Maria Luisa Testa, Maria Laura Tummino, Anna Maria Venezia, Marco Russo

**Affiliations:** 1 Institute for the Study of Nanostructured Materials, ISMN-CNR, Via Ugo La Malfa 153, 90146 Palermo, Italy; marialuisa.testa@cnr.it (M.L.T.); annamaria.venezia@cnr.it (A.M.V.); 2 Institute of Intelligent Industrial Technologies and Systems for Advanced Manufacturing, Italian National Research Council (CNR-STIIMA), Corso G. Pella 16, 13900 Biella, Italy; marialaura.tummino@stiima.cnr.it

**Keywords:** interesterification, biofuel, triacetin, sulfonic silica, grafting, silanolysis

## Abstract

In the present work, the acid-catalyzed interesterification of glyceryl trioctanoate (GTO) with ethyl acetate was investigated as a model reaction for the one-step production of biofuel and its additives. The activity of heterogeneous acid catalysts, such as silica-based propyl-sulfonic ones, was evaluated. Propyl-sulfonic groups were grafted on both amorphous and mesoporous silica oxide (SBA-15, KIT-6) using different functionalization processes and characterized by N_2_ adsorpion–desorption isotherm (BET), thermogravimetric analysis (TGA), scanning electron microscopy (SEM), attenuated total reflectance–Fourier transform infrared (ATR-FTIR) spectroscopy, and potentiometric titration. During the optimization of the reaction conditions with the most active catalyst (Am-Pr-SO_3_H), it was shown that the addition of ethanol allowed a total conversion of GTO together with 89% and 56% yield of ethyl octanoate and triacetin, respectively. The catalytic performance is strictly correlated to the catalyst features, in terms of both the acid capacity and the porous structure. Moreover, the catalytic performance is also affected by a synergistic mechanism between silanols and Pr-SO_3_H groups towards the ‘silanolysis’ of ethyl acetate. The overall results show that the presence of ethanol, the reaction time, and the amount of catalyst shifts the reaction towards the formation of the biofuel mixture composed by ethyl octanoate and triacetin.

## 1. Introduction

The progressive depletion of fossil fuels and raising awareness of their effects on climate change have boosted the research of sustainable sources of energy. Biodiesel is the second most produced biofuel and the most used in Europe, where it is blended with petroleum diesel with a percentage of 7% (*v*/*v*). To contain CO_2_ emissions, the EU Commission released the directive 2015/1513. According to this, all commercial fuels must be blended with at least 10% renewable components derived from feedstocks that do not compete with food production, such as non-edible vegetable oils, waste cooking oils, and animal fats [1]. Nowadays, biodiesel currently on the market is mainly produced by the transesterification of triglycerides of vegetable oils with methanol using a homogeneous alkaline catalyst such as NaOH and KOH [2]. Basic and acid heterogeneous catalysts and enzymes have also been widely studied, but barely used at the industrial level [3,4]. Alcoholysis of vegetable oils with homogeneous basic catalysts presents several related issues, such as the formation of glycerol and the use of homogeneous conditions. On the one hand, glycerol accounts for 10 wt% and its separation from biodiesel, as a low-grade chemical, adversely affects the costs of biodiesel production and gives off waste [5,6,7]. On the other hand, homogeneous basic catalysts, being unrecovered, produce wastewater for their neutralization and separation from the reaction mixture and hinder the use of waste oils. In fact, the presence of free fatty acid (FFA) in waste oils causes several by-products derived from saponification reactions [8].

An alternative way to overcome the drawbacks of homogeneous basic catalysis is to develop heterogeneous acid catalysis. Indeed, its use allows easy recovery and reuse of the catalyst, as well as the possibility to exploit waste oils with high content in FFA [9]. In the literature, several heterogeneous acid catalysts have been described and tested to carry out triglyceride transesterification for biodiesel production [10]. Generally, heterogeneous acid catalysts differ from each other by the type of acid sites (Lewis and Bronsted), their number, and their structure. In this respect, the catalysts can be divided into: (i) inorganic, such as metal salts, heteropolyacids, and zeolites; (ii) organic, such as sulphated polymers and resins, sulphated carbon and nanographene; and (iii) hybrid, such as alkyl or aryl sulfonic-functionalized porous oxides [11,12,13]. Regardless of the catalyst used, acid-catalyzed transesterification still has the drawback of the formation of glycerol as a by-product.

The production of biodiesel, avoiding glycerol production, can be accomplished through the interesterification reaction between triglycerides and methyl or ethyl acetate, producing in only one step fatty acids alkyl esters (FAAE) and triacetylglycerol (triacetin) [14,15]. Triacetin is a green fuel additive that can be blended with biodiesel up to 10 wt% according to the ASTM D6451 and EN 14214 standard quality. Blending with triacetin allows to increase the biodiesel yield and improves its low-temperature properties, such as cloud and pour point and its oxidation stability [16,17]. The interesterification of triglycerides has been studied using different triglyceride feedstocks [18], with and without catalysts. The process was carried out without catalysts by using supercritical methyl acetate [19,20,21,22,23], ethyl acetate [24], and higher alkyl esters [25]. Exploitation of biocatalysis was also evaluated by using both free [26] and immobilized enzymes [27,28]. Working in supercritical conditions or with enzymes as catalysts is not economically feasible from the point of view of industrial application, due to the high operative costs and long reaction time required in the case of enzymatic processes. Interesterification can be carried out in the presence of both basic and acid catalysts, which were tested in homogeneous and heterogeneous phase, either in thermal or ultrasound-assisted conditions [29,30,31,32]. Homogeneous base-catalyzed interesterification has been mainly studied due to the mild temperature and lower reaction time needed to achieve almost quantitative conversion of triglycerides and high yields of FAAE and triacetin [15,33,34]. Nevertheless, the research was principally focused on heterogeneous catalysts that can be easily removed and reused. Battistel et al. [35] carried out a wide screening of acids and bases, both in the homogeneous and heterogeneous phase, for the interesterification of tributyrin with methyl acetate. In the homogeneous phase, base catalysts (CH_3_Ona, (CH_3_)_3_COK, TBD, DBU) had the best activity, with reaction times in the order of minutes and temperature of 60–80 °C, whereas acid catalysts (methanesulfonic acid, sulphuric acid, trifluoro-acetic acid, trifluoro-methanesulfonic acid) required high temperature (130–140 °C) and longer reaction time (20 h) to obtain comparable conversions and yields. In the heterogeneous phase, the acid catalysts (Nafion SAC-13, Amberlyst-15, zirconia, zeolite β) showed comparable activity to that of the corresponding homogeneous ones, contrarily to the base catalysts (MgAl mixed oxide, ETS-10, Katalco, Pural Mg 70), whose activity was lower with respect to the homogeneous ones. Consequently, the study of heterogeneous acid catalysts has attracted the attention of researchers. Several acid heterogeneous catalysts, such as NbOPO_4_, Nb_2_O_5_, γ-Al_2_O_3_, HY, and Y zeolites, were investigated by Ribeiro et al. [36] for the interesterification of high-content FFA macaw oil with methyl acetate. The best results were achieved with γ-Al_2_O_3_ after 1 h reaction running at 250 °C (using 1:30 triglyceride to methyl acetate molar ratio and 5 wt% of catalyst loading), giving 85.2 wt% of triglyceride conversion and 52.5 wt% of total yield (FAME and triacetin).

Unsupported and γ-Al_2_O_3_-supported SnO catalysts have been investigated as heterogeneous Lewis acid catalysts for the interesterification of rapeseed oil with methyl acetate [37,38]. With an unsupported catalyst, 90% and 70% yield of FAME and triacetin, respectively, was obtained after 4 h at 210 °C by using 1:40 triglyceride to methyl acetate molar ratio and a catalyst loading of 0.69 mol/mol SnO:oil.

Tian et al. [39] tested ferric sulphate as a heterogeneous Lewis acid catalyst for the interesterification of the model oil triolein with methyl acetate, using 1:20 triglyceride to methyl acetate molar ratio, 7.5 wt% of catalyst, and methyl myristate (7.7 g/L) as co-solvent. A FAME yield of 83% after 12 h at 120 °C was achieved.

Carbonaceous-derived heterogeneous acid catalysts, as reported by Wong et al. [40,41], were tested for the interesterification of oleic acid with methyl acetate, allowing them to achieve a FAME yield of 52.3% after 8 h at 110 °C, using 1:50 triglyceride to methyl acetate molar ratio and 10 wt% of catalyst.

Among the heterogeneous catalysts investigated for the interesterification reactions, Nafion SAC-13, a commercial perfluoro-sulfonic acid polymer on porous silica, has shown the best catalytic performance [35]. This catalyst after 20 h at 130 °C gave 98% conversion of tributyrin, methyl butyrate yield of 83%, and triacetin selectivity of 60%, using 1:20 tributyrin to methyl acetate molar ratio and 5–15 wt% of catalyst [42]. Hybrid catalysts obtained by linking alkyl or aryl sulfonic groups on mesoporous silica oxide are a class of promising heterogeneous acid catalysts for the interesterification process. Usai et al. [41] tested propyl-sulfonic- and phenyl-sulfonic-acid-functionalized SBA-15 as catalysts for the interesterification of extra virgin olive oil with ethyl acetate. After 6 h at 130 °C, the propyl-sulfonic catalyst gave 6% and 0% of triglyceride conversion and FAEE yield, respectively, whereas the phenyl-sulfonic catalyst gave 20% and 19%, respectively, using in both cases 1:20 triglyceride to ethyl acetate molar ratio and 13 wt% of catalyst. This kind of catalyst has been successfully investigated in many biodiesel-related acid-catalyzed processes [43,44] including esterification [13], transesterification [45], and acetylation [46]. Hybrid catalysts have the peculiarity of acting as “homogeneous supported” catalysts. Flexible organic pendants, especially for alkyl sulfonate chains, could easily approach carbonyl esters, improving process efficiency. Sulfonated mesoporous oxide can be easily synthesized by a one-pot process during sol–gel condensation, or by grafting of the mesoporous oxide already formed. These materials are characterized by high thermal stability and tunable textural property. As a matter of fact, surface area, pore size, pore volume, and acidity, as well as amount and strength of acid sites, can be modified to some extent in order to obtain tailored catalysts.

**Figure 1:**
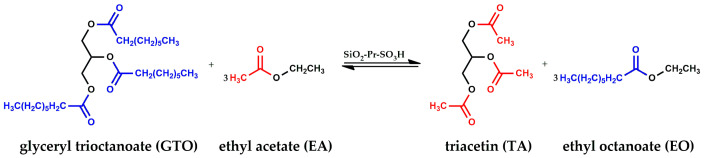
Interesterification reaction of glyceryl trioctanoate (GTO) with ethyl acetate (EA).

On these premises, and aiming to optimize the FAEE production process, hybrid Pr-SO_3_H silica catalysts were investigated for the interesterification of glyceryl trioctanoate (GTO) with ethyl acetate (EA) to produce in only one step ethyl octanoate (EO) and its additive triacetin (TA) (Figure 1).

Both amorphous and mesoporous silica (SBA, KIT) supports were functionalized with sulfonic groups by one-pot synthesis or by post-modification in thermal or hydrothermal conditions. For the sake of clarity, some of these catalysts were recently tested in MW-assisted solketal production, as an additive of biofuel, in the framework of biodiesel research field [47]. All the catalysts were fully characterized by N_2_ adsorption–desorption isotherm (BET), thermogravimetric analysis (TGA), scanning electron microscopy (SEM), attenuated total reflectance–Fourier transform infrared (ATR-FTIR) spectroscopy, and potentiometric titration. Interesterification products were analyzed by gas chromatography coupled to a mass analyser (GC–MS). The role of a synergistic effect of ethanol [48] and silanol group in shifting the equilibrium towards the formation of triacetin with respect to mono- and diglyceride intermediates was investigated. The catalytic activity was correlated to the acid and textural properties of the catalysts and these, in turn, to the route used for their synthesis.

## 2. Materials and Methods

### 2.1. Materials

Tetraethyl orthosilicate (TEOS, 98%), 3-mercaptopropyltrimethoxy silane (MPTMS, 95%), hydrogen peroxide solution (35%), glyceryl trioctanoate (≥99%), ethyl octanoate (≥99%), triacetin (≥99%) (also used as standard), and anhydrous ethyl acetate were purchased from Sigma Aldrich (Milano, Italy); absolute ethanol was purchased from VWR (Milano, Italy).

### 2.2. Catalysts Synthesis

#### 2.2.1. Mesoporous Silica Supports

Two types of silica, henceforth referred to as SBA-15 and KIT-6, were synthesized by templated sol–gel techniques according to previously described procedures.

For the synthesis of SBA-15 [49], 16.2 g of Pluronic 123^®^ was dissolved in a mixture of 294 mL of deionized water and 19.8 mL HCl (12.17 M) and left stirring overnight at 35 °C in a polypropylene bottle. To this solution, 32.1 g (0.154 mol) of TEOS was added and left stirring at 35 °C for 24 h, then it was aged at 100 °C for 24 h without stirring in the sealed bottle. The obtained wet gel was filtered under vacuum and washed repeatedly with hot deionized water (40–60 °C), in order to remove most of the template agent, and then with ethanol. The resulting solid product was calcined in air at 500 °C for 5 h (heating ramp of 1 °C/min).

For the synthesis of the KIT-6 [50], 6 g of P123^®^ was dissolved in a mixture of 217 mL deionized water, 11.8 mL HCl (12.17 M), and 7.4 mL of butanol and stirred at 35 °C for 1 h in a polypropylene bottle. Then, 13 g (0.062 mol) of TEOS was added and left to stir overnight. The mixture was aged at 100 °C for 24 h without stirring in the sealed bottle. The obtained wet gel was filtered under vacuum and washed with hot water and ethanol. The resulting solid product was calcined in air at 550 °C for 6 h (heating ramp of 2 °C/min).

#### 2.2.2. Propyl-Sulfonic (Pr-SO_3_H) Catalysts

**Figure 2:**
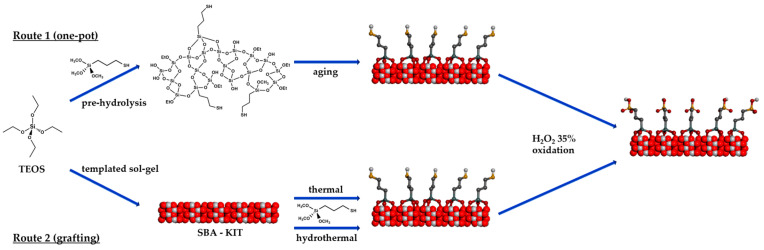
Synthetic routes for the preparation of catalysts by (route 1) one-pot and (route 2) grafting technique.

Two different procedures, one-pot synthesis (route 1) and post-modification in thermal and hydrothermal conditions (route 2), as represented in Figure 2, were carried out for the synthesis of Pr-SO_3_H silica.

One-pot sol–gel synthesis was used to prepare a propyl-sulfonic amorphous silica (Am-Pr-SO_3_H), according to previously described procedures [51]. Grafting was used to functionalize mesoporous support SBA-15 and KIT-6, exploiting both thermal (SBA-Pr-SO_3_H, KIT-Pr-SO_3_H) and hydrothermal condition (SBA-Pr-SO_3_H_HT, KIT-Pr-SO_3_H_HT) according to previously described procedures [47,51,52].

For the synthesis of Am-Pr-SO_3_H [51], 7.05 g (0.034 mol) of TEOS was dissolved in 5 mL of ethanol and stirred at 45 °C for 15 min. Then, 5 mL aqueous acetic acid solution at pH 5 was added to the mixture. This was followed by the addition of 10% mol/mol of 3-mercaptopropyltrimethoxy silane (MPTMS) and hydrogen peroxide (35% *w*/*v* solid:liquid ratio of 1:18). The solution was heated to 80 °C and left at this temperature until the formation of the gel occurred. The obtained wet gel was dried at 110 °C overnight.

For the synthesis of SBA-Pr-SO_3_H, KIT-Pr-SO_3_H [51], following a typical synthesis, 2 g of SBA or KIT silica was suspended in dry ethanol (35 mL) and 1 mL (5.38 mmol) of MPTMS was added. The mixture was left refluxing overnight. The obtained product was filtered, washed with ethanol, and dried at 120 °C overnight. Thereafter, the recovered products were suspended in methanol. Then, 2 mL of 35% hydrogen peroxide solution was added to oxidize the mercaptopropyl groups. The mixture was left stirring at room temperature for 24 h. After filtration, the solid was dried at 80 °C overnight.

For the synthesis of SBA-Pr-SO_3_H_HT, KIT-Pr-SO_3_H_HT [47], following a typical synthesis, 1 g of support, and 1mL (5.38 mmol) of MPTMS was mixed in a PTFE vessel by adding methanol dropwise up to obtain a homogeneous paste. The PTFE vessel was inserted in a steel autoclave which was heated at 180 °C for 18 h. The obtained product was washed with distilled water and ethanol, and dried at 120 °C overnight. Thereafter, the recovered products were suspended in methanol and 2 mL of 35% hydrogen peroxide solution was added to oxidize the mercaptopropyl groups. The mixture was left stirring at room temperature for 24 h. After filtration, the solid was dried at 80 °C overnight.

### 2.3. Catalyst Characterization

The textural properties were obtained by N_2_ adsorption/desorption isotherms using a Micromeritics ASAP2020 Plus 1.03 (Micromeritics, Ottawa, ON, Canada). Before the analyses, samples were outgassed at 100 °C for 4 h. The fully computerized analysis of the N_2_ adsorption isotherm at −196 °C in the standard pressure range 0.05–0.3 p/p0 allowed us to obtain, through Brunauer–Emmett–Teller (BET) model [53], the specific surface areas (SSA) of the samples. The micropore area was evaluated using the t-plot method. The total pore volume (V_p_) and average pore diameter (d_p_) were evaluated on the basis of the amount of nitrogen adsorbed at a relative pressure of 0.998, while mesopore size distribution values and mesopore volumes were calculated by applying the Barrett–Joyner–Halenda (BJH) model in the range of p/p0 of 0.1–0.98.

The thermogravimetric analyses of the samples were performed in air using the TGA 1 Star System of Mettler Toledo (Mettler Toledo, Schwerzenbach, Switzerland). About 10 mg of sample was heated from room temperature to 100 °C, left at this temperature for 30 min and then heated to 1000 °C at a rate of 10 °C/min in 40 mL/min of air.

The acid capacity of catalysts was determined by titration: 0.1 g of solid was added to 10 mL of 1 M NaCl aqueous solution and left to equilibrate to allow cations exchange. The resulting suspension was titrated by dropwise addition of 0.01 M NaOH solution using a pH-meter Mettler-Toledo 8603 to detect the equivalence point.

SEM measurements were performed to investigate the sample morphology using an EVO10 Scanning Electron Microscope (SEM, Carl Zeiss Microscopy GmbH, Oberkochen, Germany) with an acceleration voltage of 20 kV. The samples were sputter-coated with a 20 nm-thick gold layer in rarefied argon, using a Quorum SC7620 Sputter Coater (Lewes, UK).

ATR-FTIR spectra were recorded with a Thermo Nicolet iZ10 spectrometer (Milan, Italy) equipped with a Smart Endurance TM (ZnSe crystal) in the range 4000–650 cm^−1^ with 32 scans and 4 cm^−1^ band resolution.

### 2.4. Interesterification Reaction and Products Analysis

Following a typical test, the reaction mixture composition was 1.172 mL of ethyl acetate (EA, 1.057 g, 12 mmol), 197 µL of glyceryl trioctanoate (GTO, 188.3 mg, 0.4 mmol, molar ratio EA:GTO = 30), a catalyst loading of 10 mol% of acid groups with respect to starting GTO moles (10 mol%H^+^/mol GTO, for most of the tests), and 23.7 µL of ethanol (E, 18.7 mg, 0.4 mmol, molar ratio E:GTO = 1). The test was performed for different times under stirring at 120 °C.

Catalytic tests were carried out in screw cup glass vials with a volume of 7 mL using a heating magnetic stirrer plate VELP Scientifica AREX-6 Digital PRO equipped with aluminium blocks with an insert to accommodate vials.

After the end of a test, the vial was removed from the heating systems and cooled down at room temperature by inserting the vials in a cold aluminium block. During the few minutes of cooling, most of the catalyst settled down and the supernatant solution was recovered and filtered by using 0.45 µm PTFE filter in order to remove the last trace of catalyst. The filtered reaction solution was transferred to a clean vial to store it at 4 °C. A small amount of the solution (326 µL) was used to prepare a set of diluted solutions, the last one of it, with an approximate concentration of 50 ppm *v*/*v* (with respect to the starting GTO), was used to quantify the reaction product and GTO conversion by gas chromatography.

In particular, 0.7 µL of the diluted solution was analyzed with a Gas Chromatograph Shimadzu GC-17A equipped with a Phenomenex Zebron ZB-5 capillary column (length 30 m, external diameter 0.25 mm, internal diameter 0.25 µm) using a single quadrupole detector Shimadzu QP5050A GC-MS. The GC oven heating was set at 60 °C for the first 3 min, then the temperature was increased with a heating ramp of 10 °C/min up to 300 °C and this temperature was maintained for 5 min. The injector and interface temperature were set at 280 °C. Helium was used as carrier gas with a flow rate of 1.7 mL/min, a total flow of 35 mL/min, and a split ratio of 1:18.

The amount of unconverted glyceryl trioctanoate (GTO), ethyl octanoate (EO), and triacetin (TA) were evaluated by using calibration curves obtained by analysing solution of known concentration of each pure compound.

Conversion of GTO (1), yield of EO (2), and yield of TA (3) were obtained by the following equations:

(1) $$\chi_{\%GTO}=\left(\frac{n_{iGTO}-n_{fGTO}}{n_{iGTO}}\right)\times 100$$

(2) $$Y_{\%EO}=\left(\frac{n_{EO}}{3\cdot n_{iGTO}}\right)\times 100$$

(3) $$Y_{\%TA}=\left(\frac{n_{TA}}{n_{iGTO}}\right)\times 100$$

where n_iGTO_, n_fGTO_, n_EO_, and n_TA_, are initial and final moles of GTO, obtained moles of EO and moles of TA, respectively.

**Figure 3:**
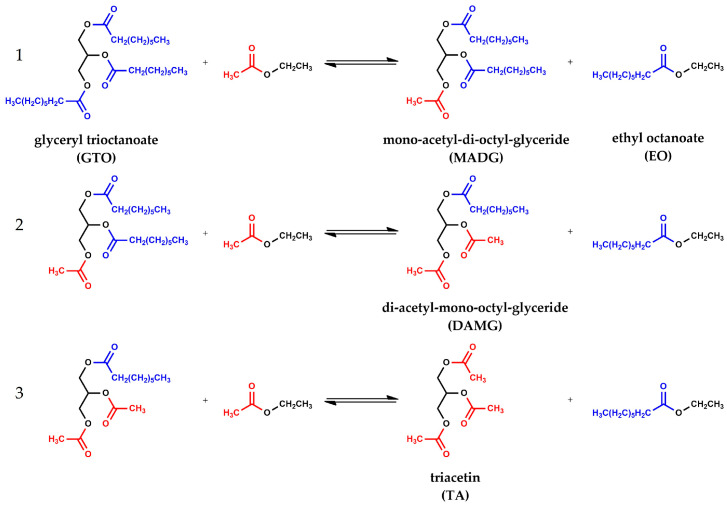
Consecutive interesterification steps between: (**1**) glyceryl trioctanoate (GTO) and ethyl acetate (EA) to give mono-acetyl-di-octyl-glyceride (MADG) and ethyl octanoate (EO); (**2**) MADG and EA to give di-acetyl-mono-octyl-glyceride (DAMG) and EO; (**3**) DAMG and EA to give triacetin (TA) and EO.

For the sake of clarity, it is worth noting that interesterification is a multi-step process in which the substitution of the acyl moieties with acetyl groups is not a simultaneous process, but rather a process consisting of three consecutive and reversible single steps (Figure 3).

**Figure 4:**
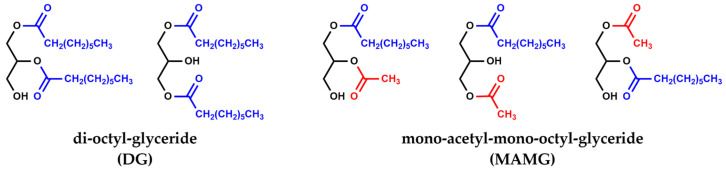
Isomers of di-octyl-diglyceride (DG) and isomers of mono-acetyl-mono-octyl-glyceride (MAMG).

This means that to obtain TA, the formation of two intermediates is necessary, mono acetyl-di-octyl-diglycerides (MADG) and di-acetyl-mono-octyl-glycerides (DAMG), in which acyl moiety is replaced by one and two acetyl groups, respectively. Then, it is also important to ascertain the amount of MADG and DAMG present in the reaction mixture. Furthermore, intermediates of partial transesterification as di-octyl-glyceride (DG) and mono-acetyl-mono-octyl-glyceride (MAMG) are also detected (Figure 4).

For the evaluation of DGs and MAMGs, an empirical calibration coefficient was determined, considering the instrumental response factor as a function of TA and GTO concentration [35]. Then, the yield of each intermediate was calculated by Equation (3).

The obtained quantities were verified by calculating GTO conversion and EO yield with the following equations:

X_%GTO_ = Y_%MADG_ + Y_%DAMG_ + Y_%MAMG_ + Y_%DG_ + Y_%TA_ (4)

(5) $$Y_{\%\mathrm{EO}}=\left(\frac{3\cdot n_{\mathrm{TA}}+2\cdot n_{\mathrm{DAMG}}+2\cdot n_{\mathrm{MAMG}}+n_{\mathrm{MADG}}+n_{\mathrm{DG}}}{3\cdot n_{\mathrm{iGTO}}}\right)\times 100$$

GTO conversion (4) was calculated as sum of the yields of products with glycerin skeleton, while EO yield (5) was calculated taking into account the corresponding moles of EO derived from each intermediate and TA. Comparison with GTO conversion and EO yield calculated with calibration curve shows a mean percentage difference in the order of ±5%, corroborating the validity of this determination.

## 3. Results and Discussion

### 3.1. Catalyst Characterization

Table 1 reports the obtained results along with the data related to pristine supports, SBA-15 and KIT-6, used in the grafting procedure. Further insight into the crystal structure of pristine SBA-15 and KIT-6 was achieved by means of XRD analyses and is reported in a previous work [47].

**Figure 5:**
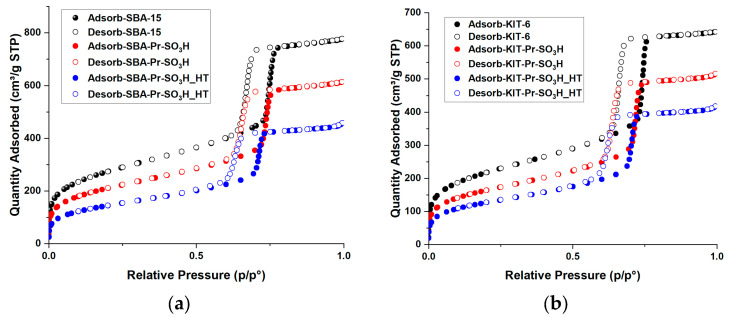

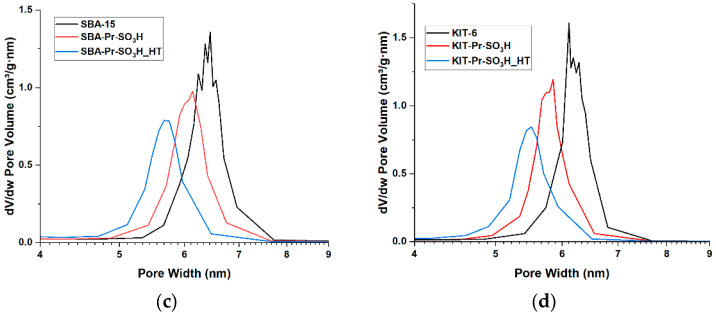
N_2_ absorption–desorption isotherm of: (**a**) SBA-15-based materials; (**b**) KIT-6-based materials; (**c**) pore size distribution of SBA-15-based materials; (**d**) pore size distribution of KIT-6-based materials.

The discussion on the N_2_ adsorption–desorption isotherms of pristine supports and related sulfonic derivatives obtained both by thermal or hydrothermal grafting is herein deepened with respect to previous work to better define the relationships between catalyst characteristics and performances. The isotherms showed a type IV shape with a H1-type hysteresis loop (Figure 5a,b) [54]. These isotherms are typical of mesoporous materials with a high surface area and the presence of cylindrical channels arranged in a hexagonal honeycomb-like structure. After thermal grafting with propyl-sulfonic groups, an evident reduction in the amount of nitrogen adsorbed is observed as a consequence of the reduction in both surface area and pore volume.

Surface area reduction of 23% and 47% is observed for SBA-15-based catalysts after thermal and hydrothermal grafting, respectively. Almost the same trend is observed for pore volume, with a reduction of 19% and 39%, respectively. KIT-6-based catalysts show the same behaviour with 25% and 42% of surface area reduction, 17% and 32% of pore volume reduction after thermal and hydrothermal grafting, respectively.

This trend is mirrored by the reduction in the pore size distribution (Figure 5c,d), which, in the pristine support, is centered between 6.0–6.7 nm and, after thermal grafting, slightly shifts to 5.7–6.4 nm and 5.5–6.1 nm for catalysts (4) and (6), respectively. Also in this case, the hydrothermal grafting has a more marked effect on pore size reduction, whose distribution shifts and narrows to 5.4–6.0 nm and 5.5–5.9 nm for catalysts (5) and (7), respectively. Progressive reduction in pore volume on passing from pristine to functionalized materials is also revealed. It is also worth noting the effect of functionalization on the contribution of micropore-related area to the total surface area. Indeed, in the case of thermal grafting, the micropore area is partially reduced from 126 m^2^g^−1^ to 80 m^2^g^−1^ and from 104 m^2^g^−1^ to 86 m^2^g^−1^ for SBA-15 materials and for KIT-6 materials, respectively. Similarly, a noticeable reduction in the micropore area after hydrothermal grafting is observed up to 38 m^2^g^−1^ and 54 m^2^g^−1^ for catalysts (5) and (7), respectively.

Everything considered, thermal grafting results in a quite uniform functionalization within the channels of the mesoporous structure, causing only partial obstruction of the micropores. However, when grafting is carried out in hydrothermal conditions, a higher micropores blockage occurs. The latter effect could be due to a higher degree of functionalization or to a modification of the support structure under harsh hydrothermal conditions.

**Figure 6:**
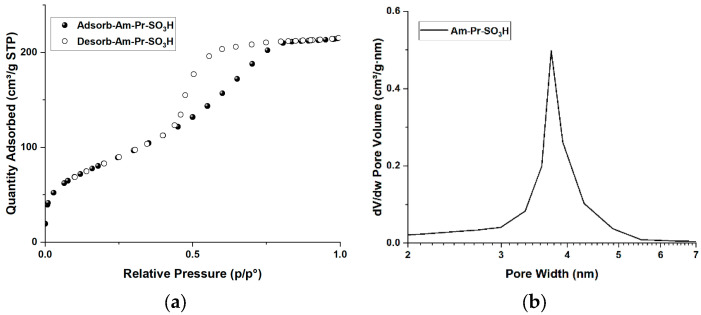
Am-Pr-SO_3_H textural properties: (**a**) nitrogen absorption–desorption isotherm; (**b**) pore size distribution.

The N_2_ adsorption–desorption isotherms of Am-Pr-SO_3_H catalyst show a type IV shape with an H_2_-type hysteresis loop, which is typical of mesoporous materials with a low surface area and the presence of pores with a narrow neck and wide body or ink-bottle shape (Figure 6a) [54,55]. Pore size distribution (Figure 6b) is quite sharp and centered between 3.6 and 3.9 nm, whereas pore volume shows the lowest value with respect to the functionalized catalysts. This means that pores and micropores do not have a marked effect on surface area.

**Figure 7:**
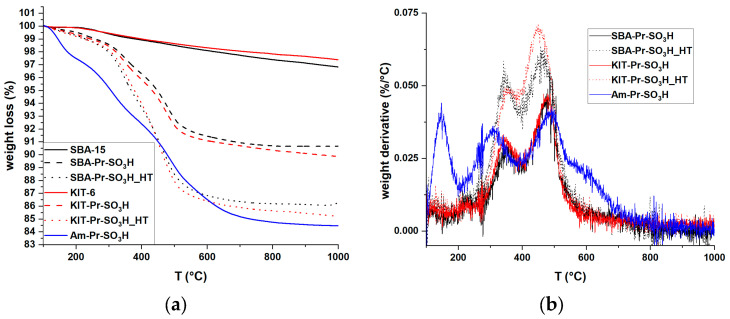
(**a**) Thermogravimetric analysis of catalysts; (**b**) 1st derivative of weight loss.

According to the TGA results shown in Figure 7, the two pristine supports behave similarly, with a continuous loss of weight with temperature, likely due to a gradual removal of water from the condensation of silanol groups [56]. As given in Table 1, similar weight losses due to the removal of the sulfonic groups are observed for the hybrid catalysts, with larger losses in the case of the hydrothermal grafting. As clearly shown by the first derivative curves of both types of catalysts, the weight losses occur in correspondence with two distinct temperatures (ca. 350 and ca. 470 °C), accounting for two favored sites of quite regular placement of propyl-sulfonic groups on the support surface. On the contrary, in the case of Am-Pr-SO_3_H, three weight losses occur at ca. 150, 310, and 480 °C, and in a wider range at ca. 600 °C. The presence of these losses is in accord with the irregular structure of the material and the subsequent irregular distribution of propyl-sulfonic groups. In addition, the presence of a weight loss at ca. 150 °C is related to the presence of crystallization water, confirming once more the main presence on superficial propyl-sulfonic groups, which can easily interact with moisture [49].

As observed in Table 1, a correlation between the TGA-derived loading of the functional groups and the catalyst acidity can be envisaged. Indeed, an increase in the acidity corresponds to an increase of the functionalization.

However, larger acidity is observed in the KIT-6-based catalysts with respect to SBA-15-based ones. These behaviors could be explained by keeping into account the aforementioned variations in the micropore area of these materials upon functionalization.

In this regard, SBA-15 shows a higher variation in micropore surface area with 37% and 70% reduction for thermal and hydrothermal grafting, respectively, whereas KIT-6 experiences 17% and 48% reduction in micropore surface area for the two types of grafting, respectively.

For SBA-15, the micropore functionalization seems more important with respect to KIT-6. The lower acidity can then be explained by two possible effects: (i) sulfonic acid groups inside the pores are hardly approached by the titrating agent, (ii) the proximity of thiol groups within pores may lead to the formation of disulfide bridges [49], resulting in the reduction in sulfonic acid groups.

Regarding the effect of the grafting method used, hydrothermal grafting allows for achieving catalysts with higher loading [47]. This finding is also corroborated by the greater values of acidity with respect to catalysts synthesized by thermal grafting. Nevertheless, catalysts (5) and (7) still keep the already mentioned differences observed for catalysts (4) and (6) related to the different surface characteristics of the supports used.

It is worth noting that Am-Pr-SO_3_H catalyst (3) has the highest loading (15.5%) and acidity (2.0 mmol H^+^ g^−1^). These results could comply with synthesis procedure of this catalyst. Indeed, one-pot sol–gel synthesis may allow for the higher incorporation of propyl-sulfonic groups into the material structure due to the simultaneous condensation of TEOS and MPTMS. Furthermore, the direct oxidation of thiol groups with hydrogen peroxide may lead to the deconstruction of material and subsequent exposure of propyl-sulfonic groups on the material surface.

**Figure 8:**
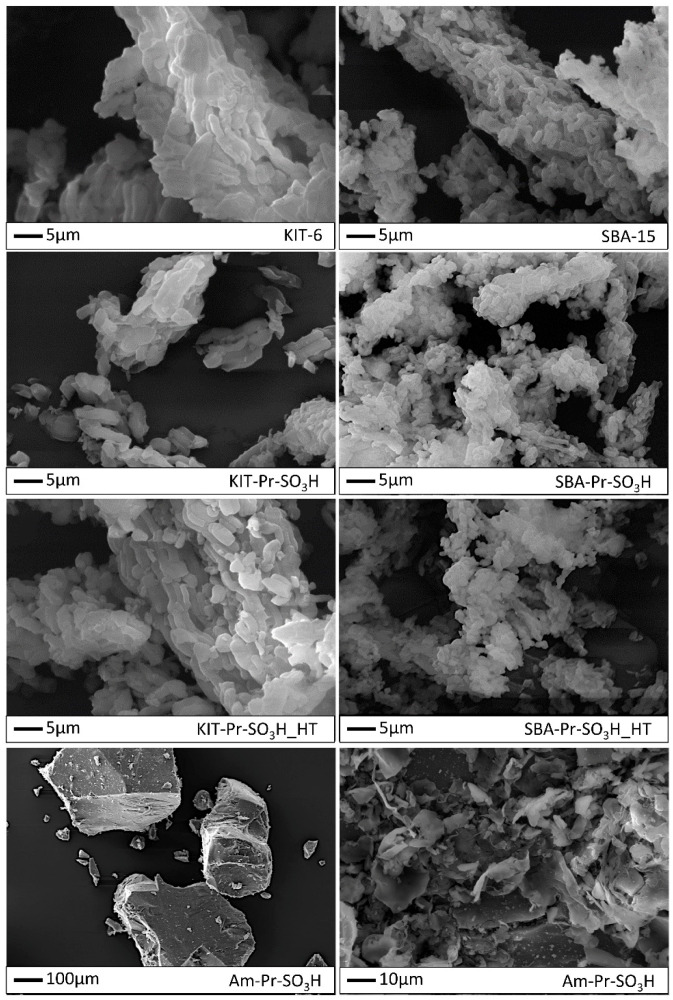
SEM images of pristine and functionalized KIT-6 and SBA-15 powders and micrographies of Am-Pr-SO_3_H powder at different magnifications.

From SEM pictures in Figure 8, KIT-based samples present different shapes: from rods to more elongated-shaped particles with squared edges, characterized by an average width of about 1.5 μm and a length up to 6 μm. SBA-based samples are shown as bunches of more regular roundish rod-like particles with an average size of about 1 μm. This is in accordance with the literature, for KIT [57] and SBA [58]. Moreover, the functionalization does not seem to influence the morphological features at this dimensional scale, nor after the hydrothermal procedure. Am-Pr-SO_3_H powder appears to consist of compacted three-dimensional large blocks measuring up to hundreds of nanometers with a flat surface, eventually covered by irregular flakes, as shown in the image at higher magnification.

**Figure 9:**
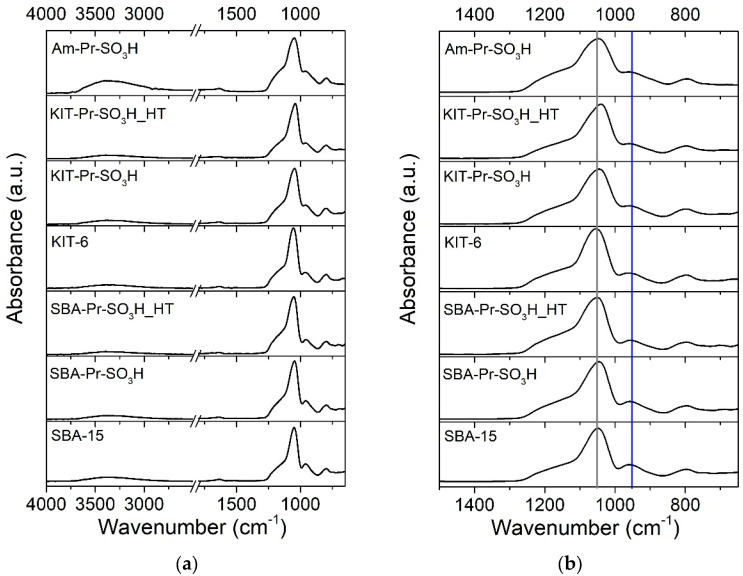
ATR-FTIR of the amorphous, KIT-6-, and SBA-15 silica-derived powders. Spectra in the entire spectral range (**a**); zoom of the infrared spectra (**b**).

In Figure 9, it is possible to see the infrared spectra of amorphous, KIT-6-, and SBA-15-derived specimens, where typical modes of silica can be highlighted. In particular, stretching vibrations of Si-O-Si are centered at 1055 cm^−1^ (including the shoulder at ca. 1180 cm^−1^) [59,60], and bending modes of Si-O-Si are visible at around 800 cm^−1^. The band at 955 cm^−1^ corresponds to the stretching of Si-OH, whereas the small signal at 1630 cm^−1^ relates to the O-H bending vibration of adsorbed water. The broad band around 3400 and 3500 cm^−1^, also appearing in the FT-IR spectra of the samples, refers to the stretching vibration of SiO-H groups interacting via H-bonding [60,61,62,63].

It is not so straightforward to detect the differences among the pristine and functionalized KIT-6- and SBA-15-based samples, due to the sensitivity of the technique (although specifically appropriate for surface analysis), but also due to the superimpositions of the sulfonic-related peaks with silica signals that make them not so evident up to certain concentrations, as has already occurred in other studies [43,49,64]. In any case, for the KIT samples after functionalization, a certain shift of the main SiO_2_ peak (1055 cm^−1^, grey line in Figure 9b) and the change in the relative intensity of the silanol peak (955 cm^−1^, blue line in Figure 9b) could be highlighted and it can be related to propyl–SO_3_H. Indeed, it has already been noticed that the interactions with functionalizing moieties can weaken the Si-OH spectral signals and modify the surface Si-O-Si original arrangement [49,65,66].

It is possible to observe some differences related to Am-Pr-SO_3_H. With respect to the other materials, the OH group signals at about 3400–3500 cm^−1^ and 1630 cm^−1^ are more intense and the shoulder at ca. 1180 cm^−1^ changes its shape, being more “fused” together with the main peak centered at 1055 cm^−1^. A similar phenomenon was revealed by Martina et al. [67], who inferred that the sulfonation of silica brought an increased intensity at 1640 and 3400 cm^−1^ due to the presence of H_2_O molecules that bound with the sulfonic acid groups. In the same paper, the modification of the 1000–1200 cm^−1^ region also occurred, as in our case. This peculiarity is coherent with the functionalization efficiency reached with Am-Pr-SO_3_H, its high acid capacity, and TGA results.

### 3.2. Preliminary Catalytic Tests

The catalytic efficacy of the catalysts for the interesterification of glyceryl triocatanoate (GTO) with ethyl acetate (EA) was assayed by preliminary screening tests. These were performed for 18 h at 120 °C by using a 1:30 EA: GTO molar ratio. In these preliminary tests, in order to trigger the reaction, ethanol was added in 1:1 molar ratio with respect to GTO. The catalyst amount was set, taking into account the different loading of acidic groups in the catalysts (see Table 1), maintaining, for the experiments, a 10 mol% of acid groups with respect to starting GTO moles. In such a way, the same amount of acid groups for each catalyst was used by varying the weight of the catalyst.

**Figure 10:**
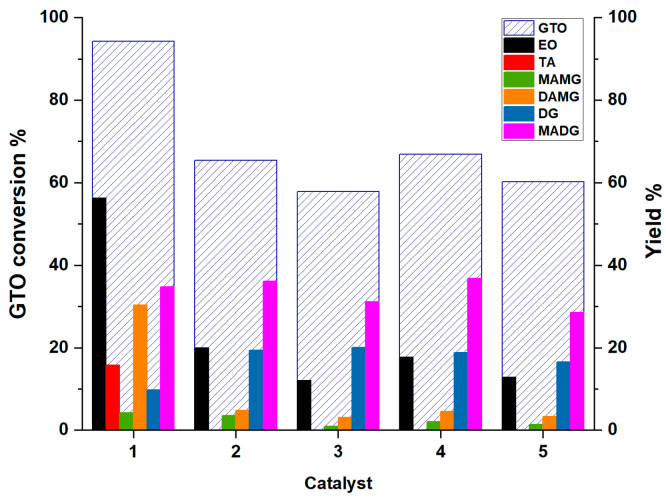
Results of catalytic tests with 10 mol%H+/mol GTO of catalyst, molar ratio E:GTO = 1, EA:GTO = 30, T = 120 °C, t = 18 h. Catalyst label: 1 (Am-Pr-SO_3_H), 2 (SBA-Pr-SO_3_H), 3 (SBA-Pr-SO_3_H-HT), 4 (KIT-Pr-SO_3_H), 5 (KIT-Pr-SO_3_H_HT).

After each test, the catalyst was filtered and the reaction mixtures were analyzed by GC–MS. The results obtained in terms of GTO conversion and product yields are summarized in Figure 10. For the sake of comparison, the catalytic activity of pristine SBA-15 and KIT-6 was also assayed, showing no catalytic effect.

It is worth noting that only with Am-Pr-SO_3_H (1), triacetin (TA) product is observed. Products of partial interesterification such as di-acetyl-mono-octyl-glycerides (DAMG), mono acetyl-di-octyl-diglycerides (MADG), and intermediates of partial transesterification such as di-octyl-glyceride (DG) and mono-acetyl-mono-octyl-glyceride (MAMG), are detected for each catalyst.

Going into details, the catalyst Am-Pr-SO_3_H shows the best activity with 94.2% of GTO conversion, and a 56.2% and 15.7% yield of EO and TA, respectively. Moreover, the Am-Pr-SO_3_H catalyst achieves the highest ratio among intermediates of interesterification (MADG and DAMG) and intermediates of transesterification (DG and MAMG). This highlights that the process is shifted more towards the interesterification products with respect to other catalysts, as confirmed by the highest amount of DAMG and the presence of TA. SBA-15- and KIT-6-based catalysts show lower GTO conversion and EO yield. These catalysts have almost the same activity both for GTO conversion and EO yield, with just a little difference between materials obtained by thermal synthesis and those obtained by hydrothermal (HT) conditions. In fact, SBA-Pr-SO_3_H (2) and KIT-Pr-SO_3_H (4) show a GTO conversion of 65.4 and 66.9% and an EO yield of 20 and 17.7%, respectively. On the other hand, SBA-Pr-SO_3_H_HT (3) and KIT-Pr-SO_3_H_HT (5) show slightly lower GTO conversions of 57.8 and 60.3% and EO yields of 12 and 12.8%, respectively. As concerns the formed intermediates, it is necessary to point out the mechanism of the interesterification process. Interesterification of GTO with EA is a three consecutive steps process [15,26,33,48] (see Figure 3 for chemical structure details) that can be represented as follow:

GTO + EA ↔ MADG + EO(R1)

MADG + EA ↔ DAMG + EO(R2)

DAMG + EA ↔ TA + EO(R3)

**Figure 11:**
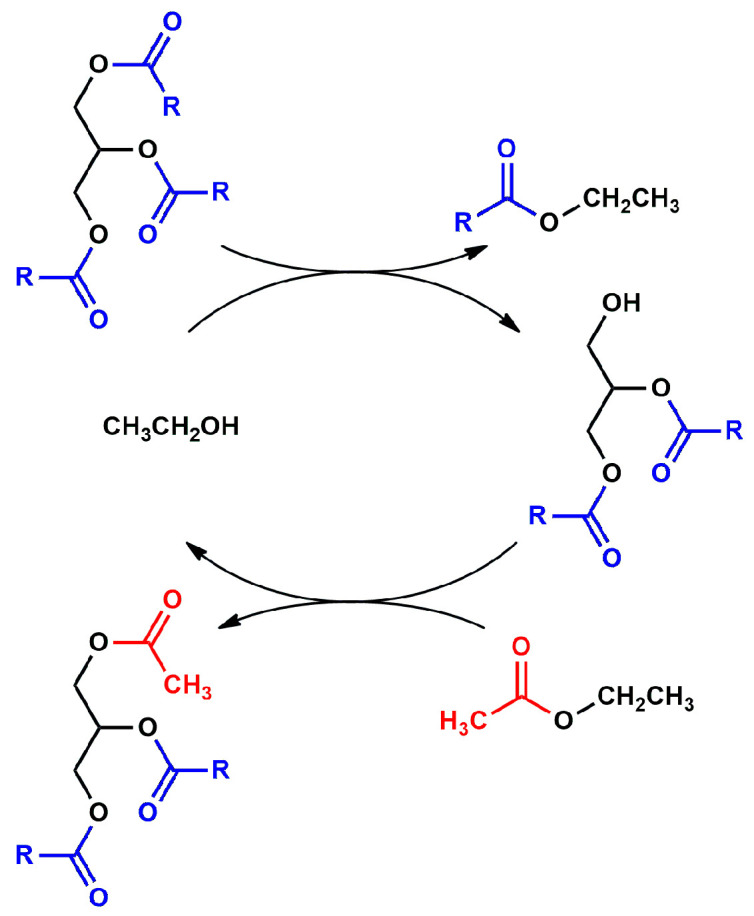
Representation of each interesterification step as a pair or transesterification reactions.

As reported by Casas et al. [33], each step can be considered as a couple of transesterification reactions. Considering the first step (R1) of the sequence, a molecule of ethanol (E) provides the transesterification of GTO to give a molecule of EO and one of DG (R4). The latter reacts with ethyl acetate (EA) to give MADG and the release of a new molecule of ethanol (R5), which can be re-involved in the sequence of reactions starting from MADG and DAMG. Figure 11 reports a generic representation of this consecutive process.

GTO + E ↔ DG + EO(R4)

DG + EA ↔ MADG + E(R5)

MADG + E ↔ MAMG + EO(R6)

MAMG + EA ↔ DAMG + E(R7)

DAMG + E ↔ DAG + EO(R8)

DAG + EA ↔ TA + E(R9)

The described mechanism justifies the addition of ethanol in a catalytic amount and takes account of the intermediates, except for di-acetyl-glycerol (DAG), which has never been detected.

**Figure 12:**
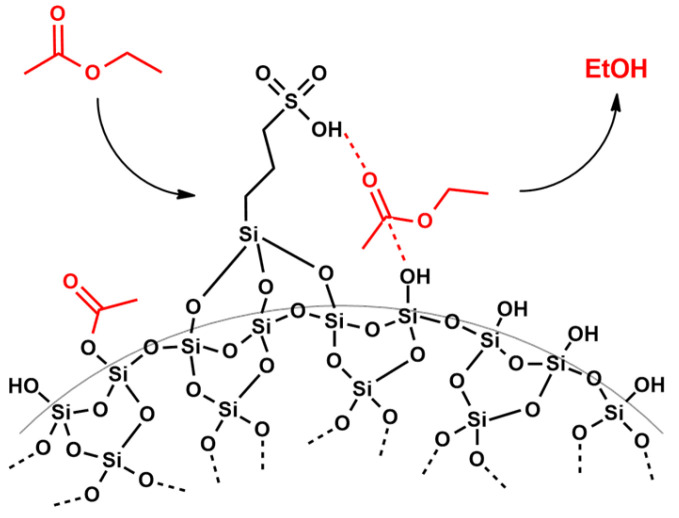
Representation of ethyl acetate hydrolysis by silica catalysts.

Moreover, considering the structure of sulfonic silica catalysts, it is possible to hypothesize that sulfonic and silanol groups can synergistically achieve the silanolysis of ethyl acetate, providing further ethanol, as also confirmed by Dyker [68] (see Figure 12).

It is possible to explain the lower catalytic activity of sulfonic SBA-15- and KIT-6-based catalysts, with respect to sulfonic amorphous silica, in light of their structural and surface properties.

These catalysts are, in fact, characterized by the presence of meso- and micro-pores, which heavily contribute to the surface area and to the degree of functionalization. This may, in turn, reduce the accessibility of GTO in the pores and, thus, the possibility of approaching with sulfonic acid groups, also due to pore volume reduction after grafting (see Table 1). This effect is more pronounced for catalysts obtained by hydrothermal grafting, which, therefore, show lower catalytic activity with respect to the thermal ones. This can also be justified by taking into account the presence of disulfide bridges, which can further hinder GTO diffusion through the channels. Another aspect to be considered is that the high surface area of mesoporous catalysts, which account for a higher quantity of silanol groups, which can increase the hydrolysis of ethyl acetate. This increases the ethanol amount but leaves acetyl groups on the silica surface. Thus, more transesterification products are formed, but they cannot readily interact with the acid and acetyl groups to be converted to acetyl glycerides and then to TA. Am-Pr-SO_3_H shows the best catalytic activity due to its amorphous structure and, thus, the better access for GTO to catalytic acid sites present on “external” catalyst surface.

Another consideration that deserves attention is the weight percentage of the catalysts used. Fixing the molar content of acid groups to 10% means using a different weight percentage of each catalyst according to its acidity. For each mole of GTO, the corresponding wt% of heterogeneous catalyst are 11%, 53%, 38%, 48%, and 32% for Am-Pr-SO_3_H, SBA-Pr-SO_3_H, SBA-Pr-SO_3_H_HT, KIT-Pr-SO_3_H, and KIT-Pr-SO_3_H_HT, respectively. It is evident how for SBA-15- and KIT-6-based materials, which present lower acidity, a higher amount of catalyst is used with respect to the Am-Pr-SO_3_H catalyst. In consideration of the lower activity/catalyst amount ratio, these catalysts were not considered for any further investigation. On the contrary, the promising catalytic activity of Am-Pr-SO_3_H was then studied for the optimization of the reaction parameters as for the effect of reaction time, ethanol addition, and catalyst amount.

### 3.3. Effect of Time and Ethanol on the Interesterification Process

The effect of reaction time was investigated by keeping fixed other reaction parameters (T = 120 °C; molar ratio EA:GTO = 30; catalyst 10 mol%H^+^/mol GTO). In addition, the kinetics was replicated with and without ethanol to assess its effect on reaction rate, yield, and conversion.

**Figure 13:**
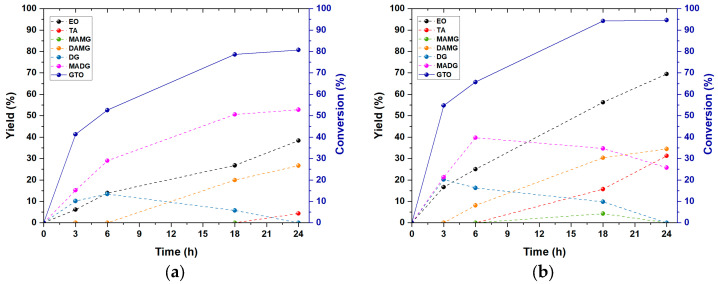
Kinetics of interesterification of GTO with EA by using Am-Pr-SO_3_H as catalyst: (**a**) without ethanol; (**b**) with ethanol.

As seen in Figure 13, it is possible to observe that even in the absence of ethanol, there is the formation of transesterification intermediates, providing further evidence of the hydrolysis of ethyl acetate by the silica catalyst.

In the reaction without ethanol, 80.7%, 38.4%, and 4.3% yields are obtained, whereas, with ethanol, the reaction is faster and achieves higher levels of GTO conversion and yields of EC and TA, which after 24 h of reaction are 94.6%, 69.5% and 31.3%, respectively.

When the reaction is carried out without ethanol, DGs reach a maximum yield of 13.4% after 6 h and MAMG is not detected. During the reaction in ethanol, a higher presence of DG and MAMG is observed, reaching a maximum yield of 20.2% after 3 h and 4.3% after 18 h, respectively. Regarding MADG, it is the main product of the reaction without ethanol, with a yield of 52.8% after 24 h, while it reaches a maximum yield of 39.7% after 6 h in the reaction with ethanol. This difference can be ascribed to the fact that, in the presence of ethanol, GTO and MADG react with ethanol present at the beginning of the reaction to give DG (R4) and MAMG (R6), respectively. On the other hand, DAMG increases steadily in both cases, reaching 26.7% and 34.5% yield after 24 h for the reaction without and with ethanol, respectively.

According to Casas et al. [33], the conversion of DAMG to TA is the rate-determining step of the reaction. This is confirmed also here, where there is a constant accumulation of DAMG that, in the presence of ethanol, is reduced, accompanied by an increase in the amount of TA [48]. It is worth noting that di-acetylglycerol (DAG) formed and consumed in reactions (R8) and (R9) is never detected. This observation could be explained by admitting that, under the reaction conditions used, reaction (R8) has a lower reaction rate than reaction (R9). This could also explain the accumulation of DAMG and, consequently, the amount of DAG is kept low (down to the detection limit) by its conversion to TA.

Casas et al. [33] carried out the study in the presence of homogeneous basic catalysis (sodium methoxide in methanol) and methyl acetate as the acyl donor, resulting in a faster reaction with respect to that represented in this study. The base-catalyzed interesterification mechanism involves the initial removal of an α-hydrogen to carbonyl ester, by the methoxide anion, with the formation of enolic species that, following Claisen condensation, give β-ketoester and glycerolate anion intermediates, which, in turn, give rise to the methyl ester and acetyl glycerol, respectively [69]. In basic catalysis, however, in the presence of methanol, the competitive transesterification reaction can also take place via an acyl nucleophilic substitution mechanism. In fact, as reported by Liu [69], methanol can coordinate with carbonyl oxygen, favoring nucleophilic substitution by methoxide anion. Then methoxide anion can act both as a catalyst and reactive species. This explains the decrease in DAMG and TA as the amount of methanol increases observed by Casas et al. [48].

In the case of acid-catalyzed interesterification, a plausible reaction mechanism involves a double transesterification reaction for each of the interesterification steps. In particular, the acid catalyst activates carbonyl, which undergoes nucleophilic attack by ethanol to form a FAEE molecule and a diglyceride. The latter has a free hydroxyl that acts as a nucleophile and attacks the carbonyl, also activated by the acid catalyst, of an ethyl acetate molecule, giving MADG. The same pair of transesterification reactions is repeated in sequence for subsequent steps until TA is formed (reactions (R4)–(R9)). Thus, in the case of acid-catalyzed interesterification, ethanol triggers the interesterification process and shifts the equilibrium towards the formation of acetyl glycerides. In this case, the competition between interesterification and transesterification is not so pronounced because reactions share a common step and the same type of reaction mechanism. Further proof is provided by the formation of the interesterification and transesterification intermediates in the absence of ethanol.

### 3.4. Effect of Catalyst Loading on Conversion and Yields

**Figure 14:**
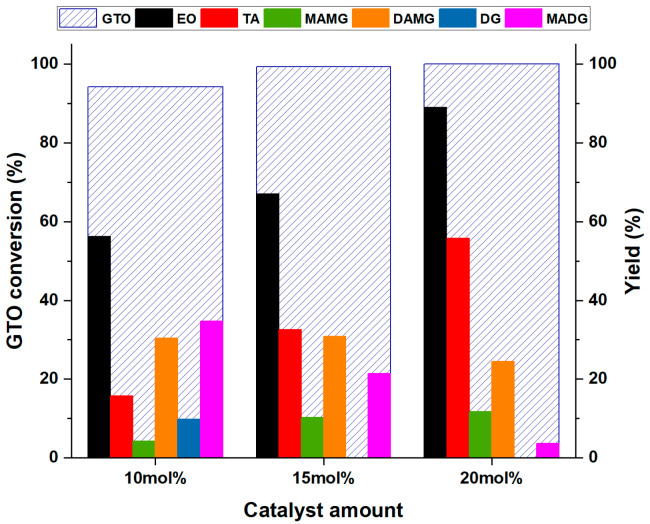
Effect of catalyst amount on interesterification of GTO with Am-Pr-SO_3_H in presence of 1 mol% of ethanol, T = 120 °C, t = 18 h.

Finally, the effect of the amount of catalyst on the interesterification of GTO was tested. The reactions were carried out at 120 °C for 18 h with a catalyst amount of 15 mol% and 20 mol% of acid groups with respect to the initial amount of GTO and in the presence of 1mol% ethanol. The data obtained were compared with those recorded in the presence of 10 mol% catalyst. Conversion of GTO, yields of EO and TA, and reaction intermediates are shown in Figure 14.

Conversion of GTO increases with the amount of catalyst, although this trend is not so evident, since 94.2% conversion is observed with 10 mol% catalyst, rising to 99.3% and 100% with 15 mol% and 20 mol% catalyst, respectively. As far as the main products EO and TA are concerned, a gradual increase in their yield with the catalyst amount is observed. The yield of EO from 56.2% increases to 67.0 and 88.9% with 15 mol% and 20 mol% catalyst, respectively. The yield of TA increases as well, from 15.7% to 32.5% and 55.7% 15 mol% and 20 mol% catalyst, respectively. Regarding the intermediates, different trends are observed. In particular, the increase in MAMG can be explained by considering a shift of the equilibrium of (R5) and (R6) as the catalyst increases, with the consequent decrease in MADG and DG, with the latter one disappearing completely with 15 mol% catalyst. The DAMG trend deserves a different consideration; although consumed by the (R8) reaction, its amount remains almost stable with 15 mol% catalyst and begins to decrease with 20 mol% catalyst amount. This confirms, once again, that the conversion of DAMG to TA is the rate-determining step.

These last results indicate that as the amount of catalyst increases, the rate of the interesterification reaction increases. It is possible that the lower amounts of catalysts do not guarantee enough sulfonic and silanol groups on the silica support that would achieve the silanolysis of ethyl acetate, providing further ethanol, as schematized in Figure 12.

In conclusion, Table 2 shows the comparison of catalytic activity between Am-Pr-SO_3_H and structurally related heterogeneous acid catalysts reported in the literature. In particular, SBA-15-supported sulfonic acid (SBA-15-Propyl-SO_3_H and SBA-15-Phenyl-SO_3_H) [42], a sulfonic polystyrene resin (Amberlyst-15^®^), and a silica-supported perfluorinated copolymer with sulfonic groups (Nafion SAC-13^®^) [35] were compared with our catalyst in terms of triglyceride conversion (X_TG_), triacetin yield (Y_TA_), and FAEE/FAME yield (Y_FAEE/FAME_) obtained under similar reaction conditions.

Am-Pr-SO_3_H shows higher activity with respect to both SBA-15-Propyl-SO_3_H and SBA-15-Phenyl-SO_3_H when the reaction is carried out with ethanol, whereas, when the reaction is carried out without ethanol addition, only a slightly lower yield of the corresponding FAEE is obtained with respect to SBA-15-Phenyl-SO_3_H. This comparison confirms once again that the use of mesoporous support can hinder the diffusion of triglycerides into the catalyst and this effect seems as high as the dimension of triglyceride increases on passing from gliceryl trioctanoate to olive oil.

With respect to Amberlyst-15, our catalyst shows a widely superior activity already after 6 h of reaction, albeit there is stronger Bronsted acidity of Amberlyst-15. Taking into account Nafion SAC-13, its activity is consistent with Am-Pr-SO_3_H, although we used a higher catalyst loading. Nevertheless, it is necessary to point out the use of a triglyceride (tributyrin) with shorter alkyl chains with respect to the GTO and methyl acetate as the acyl donor, since they are more reactive species. Another aspect to be considered is the environmental concerns related to the use of perfluorinated substances (PFAS) considered as “eternal pollutants”.

## 4. Conclusions

One-pot propyl-sulfonic amorphous silica show the best catalytic performance in the interesterification reaction of glyceryl trioctanoate (GTO) with ethyl acetate (EA), achieving 94.2% of GTO conversion, and 56.2% and 15.7% yield of ethyl octanoate (EO) and triacetin (TA), respectively. The corresponding grafted KIT-6- and SBA-15-based catalysts show lower activity in terms of both GTO conversion and EO yield, with just a little difference, ca. 5–7% for EO yield and ca. 7–8% for GTO conversion between thermal- and hydrothermal-grafted catalysts. Despite a lower specific surface area, the larger activity of the amorphous silica catalyst is attributed to its larger acid capacity as well as to the surface availability of acid groups. These features are related to a high loading of Pr-SO_3_H achieved by their efficient incorporation into the material structure due to the simultaneous condensation of TEOS and MPTMS. Performing the reaction in the presence of ethanol confirms a synergy between ethanol and the silanol group in shifting the equilibrium towards the formation of triacetin with respect to mono- and di-glyceride intermediates. The catalytic results as a function of the catalyst loading indicate that an appropriate amount of catalyst is crucial to achieve the silanolysis of the ethyl acetate, which is important for the interesterification process.

After the optimization of the reaction conditions, the best result is achieved with a catalyst loading of 20 mol%H^+^/mol GTO, giving a complete conversion of GTO, and an 89% and 56% EO and TA yield, respectively, after 18 h. The studied material could be considered promising if compared to the existing literature. This result is satisfying, also considering that among the undesired intermediates, DAMG, which accounts for 24%, can be viewed as a fuel additive as well. In order to improve the attractiveness towards an industrial application, further steps of this study foresee the optimisation of the catalyst in terms of loading of acid groups and the design of easily manageable shapes (pellet), as well as the use of waste vegetable oils.

## Figures and Tables

**Table 1:** Textural and physical properties of catalysts.

Entry	Catalyst	Acid Capacities (mmol H^+^ g^−1^) ^a^	TGA (Loading %)	BET	
				SSA (m^2^ g^−1^)	V_p_ (cm^3^ g^−1^)
1	SBA-15	-	-	988	1.09
2	KIT-6	-	-	785	0.92
3	Am-Pr-SO_3_H	2.0	15.5	306	0.34
4	SBA-Pr-SO_3_H	0.28	9.3	761	0.88
5	SBA-Pr-SO_3_H_HT	0.60	13.7	526	0.67
6	KIT-Pr-SO_3_H	0.43	10.2	589	0.76
7	KIT-Pr-SO_3_H_HT	0.72	14.8	458	0.63

^a^ It was determined by titration with NaOH 0.01 M.

**Table 2:** Catalytic activity comparison between Am-Pr-SO_3_H and structurally related catalysts reported in the literature.

Catalyst	T (°C)	t (h)	Catalyst Loading (wt%)	Acetyl Donor:TG ^d^	X_TG_ (mol%)	Y_TA_ (mol%)	Y_FAEE/FAME_ (mol%)	Ref.
Am-Pr-SO_3_H	120	6	11	30	52	0	14	This work
Am-Pr-SO_3_H ^a^	120	6	11	30	66	0	25	This work
Am-Pr-SO_3_H ^a^	120	18	22 ^b^	30	100	56	89	This work
SBA-15-Propyl-SO_3_H	130	6	13	20 ^e^	6	-	0 ^g^	[42]
SBA-15-Phenyl-SO_3_H	130	6	13	20 ^e^	20	-	19 ^g^	[42]
Amberlyst-15^®^	120	20	5–15 ^c^	20 ^f^	9	0	4 ^h^	[35]
Nafion SAC-13^®^	130	20	5–15 ^c^	20 ^f^	98	60	83 ^h^	[35]

^a^ Reaction with ethanol, molar ratio E:GTO = 1; ^b^ corresponding to 20 mol%H^+^/mol GTO; ^c^ %(*w*/*v*), exact catalyst loading not specified; ^d^ triglyceride (TG); ^e^ molar ratio ethyl acetate:olive oil = 20; ^f^ molar ratio methyl acetate:tributyrin = 20; ^g^ FAEE from olive oil; ^h^ FAME from tributyrin.

## Data Availability

The data analyzed and reported in the main text are not available in a public archive.
